# Cardiopulmonary resuscitation of adults with in-hospital cardiac arrest using the Utstein style

**DOI:** 10.5935/0103-507X.20160076

**Published:** 2016

**Authors:** Rose Mary Ferreira Lisboa da Silva, Bruna Adriene Gomes de Lima e Silva, Fábio Junior Modesto e Silva, Carlos Faria Santos Amaral

**Affiliations:** 1 Faculdade de Medicina, Universidade Federal de Minas Gerais - Belo Horizonte (MG), Brazil.

**Keywords:** Heart arrest, Cardiopulmonary resuscitation, Intensive care units, Parada cardíaca, Ressuscitação cardiopulmonar, Unidades de terapia intensiva

## Abstract

**Objective:**

The objective of this study was to analyze the clinical profile of patients with in-hospital cardiac arrest using the Utstein style.

**Methods:**

This study is an observational, prospective, longitudinal study of patients with cardiac arrest treated in intensive care units over a period of 1 year.

**Results:**

The study included 89 patients who underwent cardiopulmonary resuscitation maneuvers. The cohort was 51.6% male with a mean age 59.0 years. The episodes occurred during the daytime in 64.6% of cases. Asystole/bradyarrhythmia was the most frequent initial rhythm (42.7%). Most patients who exhibited a spontaneous return of circulation experienced recurrent cardiac arrest, especially within the first 24 hours (61.4%). The mean time elapsed between hospital admission and the occurrence of cardiac arrest was 10.3 days, the mean time between cardiac arrest and cardiopulmonary resuscitation was 0.68 min, the mean time between cardiac arrest and defibrillation was 7.1 min, and the mean duration of cardiopulmonary resuscitation was 16.3 min. Associations between gender and the duration of cardiopulmonary resuscitation (19.2 min in women versus 13.5 min in men, p = 0.02), the duration of cardiopulmonary resuscitation and the return of spontaneous circulation (10.8 min versus 30.7 min, p < 0.001) and heart disease and age (60.6 years versus 53.6, p < 0.001) were identified. The immediate survival rates after cardiac arrest, until hospital discharge and 6 months after discharge were 71%, 9% and 6%, respectively.

**Conclusions:**

The main initial rhythm detected was asystole/bradyarrhythmia; the interval between cardiac arrest and cardiopulmonary resuscitation was short, but defibrillation was delayed. Women received cardiopulmonary resuscitation for longer periods than men. The in-hospital survival rate was low.

## Abstract (pt)

**Objetivo:**

Analisar o perfil clínico de pacientes com parada cardiorrespiratória intra-hospitalar, seu atendimento e evolução, com registro baseado no estilo *Utstein*.

**Métodos:**

Estudo observacional, prospectivo e longitudinal em ambiente de terapia intensiva de pacientes com parada cardiorrespiratória incluídos durante 1 ano.

**Resultados:**

Foram 89 pacientes, com média de idade de 59,0 anos, 51,6% homens, submetidos às manobras de ressuscitação cardiopulmonar. Os episódios ocorreram no período diurno em 64,6% dos casos. A assistolia/bradiarritmia foi o ritmo inicial mais frequente (42,7%). A maior parte dos que apresentaram retorno à circulação espontânea evoluiu com parada cardiorrespiratória recorrente, principalmente nas primeiras 24 horas (61,4%). As médias dos tempos foram de 10,3 dias entre a internação e ocorrência de parada cardiorrespiratória; 0,68 minutos entre a parada cardiorrespiratória e ressuscitação cardiopulmonar; 7,1 minutos entre a parada cardiorrespiratória e a desfibrilação; 16,3 minutos de duração da ressuscitação cardiopulmonar. Houve associação entre sexo e duração da ressuscitação cardiopulmonar (19,2 minutos nas mulheres *versus* 13,5 minutos nos homens; p = 0,02), duração da ressuscitação cardiopulmonar e retorno à circulação espontânea (10,8 minutos *versus* 30,7 minutos; p < 0,001), entre cardiopatia e a idade (60,6 anos *versus* 53,6; p < 0,001). A sobrevida imediata após a parada cardiorrespiratória foi de 71% e, até a alta hospitalar e no sexto mês após a alta, de 9% e de 6%, respectivamente.

**Conclusão:**

O principal ritmo inicial detectado foi a assistolia/bradiarritmia com curto intervalo entre a parada cardiorrespiratória e a reanimação, porém com desfibrilação tardia. Mulheres apresentaram maior tempo de reanimação. Houve baixa taxa de sobrevida hospitalar.

## INTRODUCTION

Cardiac arrest (CA) is defined as the cessation of the mechanical activity of the heart and is confirmed by the absence of signs of circulation.^(1)^ In an attempt to restore the spontaneous circulation of patients and reverse CA, cardiopulmonary resuscitation (CPR) maneuvers should be performed as part of a rapid, appropriate, coordinated and standardized intervention.^(2,3)^ CA is an emergency situation, and its epidemiological data differ by setting, i.e., out-of-hospital or in-hospital.

The overall incidence of adult in-hospital CA is 1.6/1,000 admissions, and its incidence in intensive care units (ICUs) is 52%.^(4)^ Overall, the rate of survival until hospital discharge is 18.4% and ranges from 10.5% for non-shockable rhythms to 49% for shockable rhythms.^(5)^ In Brazil, a single-center study examined 536 patients who underwent CPR during a 5-year period and reported a 16.2% 1-month survival rate.^(6)^ Moreover, the rate of survival until hospital discharge was 13% in another Brazilian, multicenter study, which examined a total population of 763 patients with CA, 360 of whom experienced CA in the ICU and coronary unit.^(7)^

The Utstein style is a set of guidelines on the essential and desirable data that should be collected while treating patients with CA.^(1,8-10)^ These data allow the survival rates and treatment outcomes to be specified using variables collected and analyzed in standardized individual CPR reports of in-hospital CA patients.^(8,9)^ The Utstein-style recommendations enable the standardization of definitions and methods, which should support the validity of interpretations and findings of different studies.

However, knowledge on the profile, prognosis and outcomes of patients who underwent CPR exclusively in the in-hospital setting as well as on the use of the Utstein style-based registry for CA and CPR data is limited in Brazil.^(7,11)^

Therefore, this study aimed using the Utstein style to analyze the clinical profile and outcomes of patients who experienced in-hospital cardiac arrest.

## METHODS

This work is a prospective, observational and longitudinal study of a population of male and female patients who experienced in-hospital CA of any etiology at the coronary and adult ICU of a university institution and received CPR maneuvers. Patients who were treated from December 2011 to December 2012 were included in the study. Data were collected using medical records and Utstein-style CPR reports,^(8,9)^ which were filled out by a qualified professional involved in the care of the patient with CA. The project was approved by the Research Ethics Committee of the *Universidade Federal de Minas Gerais*, CAAE 0230.0.203.000-11. The Utstein-style CPR report had not been used prior to project approval. Patients who survived the CA and were discharged were invited to sign the Informed Consent Form before leaving the hospital. These patients were clinically evaluated at discharge using the Glasgow Coma Scale (GCS) and Cerebral Performance Category (CPC) scores. The patients were also contacted by telephone 6 months after hospital discharge, as dictated by the Utstein-style protocol.

This study used variables based on the In-Hospital Utstein style and included 3 categories, namely, patient, CA and follow-up variables.

The patient variables were gender, admission diagnosis, comorbidities, devices used prior to CA (cardiac monitor, venous access, tracheal tube, tracheostomy, mechanical ventilation, intra-arterial pressure, intra-aortic balloon, Swan-Ganz catheter, implantable cardioverter defibrillator and cardiac pacemaker), intravenous medications and GCS prior to CA.

The CA variables were its immediate cause (lethal arrhythmia, acute myocardial infarction or ischemia, hypotension, respiratory depression, metabolic abnormality, unknown cause or other cause), procedures performed (chest compression, intubation, defibrillation and artificial pacemaker placement), initial rhythm detected (ventricular fibrillation, ventricular tachycardia, pulseless electrical activity, asystole and bradycardia), time of the events (CRA, beginning of CPR, first defibrillation, first dose of intravenous medication, intubation and end of CPR), medications used (epinephrine, atropine, amiodarone, sodium bicarbonate, dopamine and dobutamine), incidence and time to return of spontaneous circulation and return of non-sustained spontaneous circulation (time period shorter than 20 minutes, longer than 20 minutes, shorter than 24 hours and longer than 24 hours).

The follow-up variables included recovery of consciousness (time from CA to recovery), other attempts at resuscitation, total length of hospital stay, length of hospital stay from CA to discharge, destination after discharge, GCS at discharge, CPC at discharge (score 1: good cerebral performance; 2: moderate cerebral disability; 3: severe cerebral disability; 4: coma or vegetative state; and 5: brain death), 6-month survival, 6-month CPC score, time from CA to death after hospital discharge (had it occurred) and time from discharge to death.

### Statistical analysis

The Statistical Package for Social Science (SPSS) software, version 14.0, was used to analyze the data. The results are expressed as numbers and percentages for categorical variables and as measures of central tendency (mean or median) and of dispersion for continuous variables. The Mann-Whitney test and the chi-squared or Fisher's test, where appropriate, were used to compare differences between continuous and categorical variables, respectively. The level of rejection of the null hypothesis was set at ≤ 0.05.

## RESULTS

A total of 452 patients experienced CA during the 1-year study period (from December 2011 to December 2012). Of these patients, 89 (19.6%) underwent CPR maneuvers and were included in the study. A total of 96 episodes of CA occurred among these patients.

The mean age of the cohort was 59 ± 17.6 years, ranging from 16 to 94 years, and 46 (51.6%) patients were male. At the time of CA, 49.4% patients were sedated, and the other 50.5% patients had GCS scores ranging from 3 to 15, with a mean of 10. Regarding the main comorbidities, 48.3% patients had systemic arterial hypertension, 28% had *diabetes mellitus*, 15.7% had heart failure, 7.8% had cancer, and 6.7% had myocardial ischemia. Furthermore, 22 patients were smokers, and 17 were alcoholics.

Data on the ICU admission diagnoses are outlined in table 1.

**Table 1:** Admission diagnoses of the 89 patients who underwent cardiopulmonary resuscitation in coronary and intensive care units

Admission diagnosis	N (%)
Acute myocardial infarction	22 (24.7)
Sepsis	15 (16.8)
Acute respiratory failure	13 (14.6)
Shock	9 (10.1)
Stroke	6 (6.7)
Pneumonia	6 (6.7)
Trauma	6 (6.7)
Pulmonary thromboembolism	4 (4.4)
Acute pulmonary edema	3 (3.3)
Neurological surgery, postoperative	3 (3.3)
Cardiac surgery, postoperative	1 (1.1)
Heart failure	1 (1.1)

**Figure 1:**
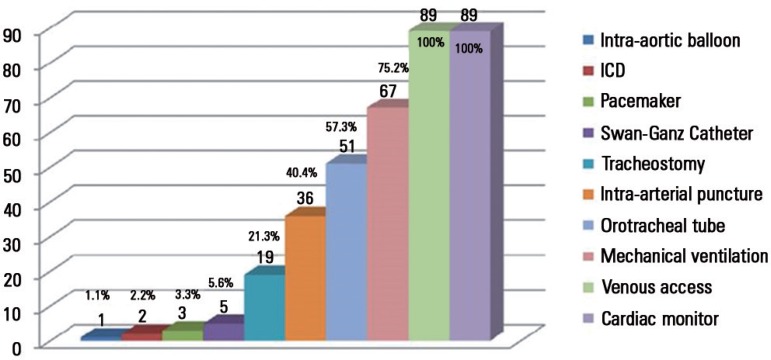
Devices used prior to cardiac arrest. ICD - implantable cardioverter-defibrillator.

All patients were undergoing cardiac monitoring and had venous access. The mean number of devices used per patient was 4. Figure 1 summarizes the data on the devices used and the number (with the percentage) of patients using the device at the time of the CA.

The assessment of the 96 episodes of CA showed that most episodes (64.6%) occurred at daytime. The initial rhythm was identified in 91 episodes of CA (considering the episodes of recurrence of CA), and asystole/bradyarrhythmia was the most frequent rhythm (42.7%). Defibrillation was performed in 32.2% episodes. Ventricular tachycardia and ventricular fibrillation were detected in 14.6% episodes at the time of the first CA and in 16.6% episodes of recurrent CA. Chest compression was not performed in 2 episodes of CPR because the patients exhibited a return of spontaneous circulation with defibrillation shock alone. No medication was administered during CPR in 5.2% episodes because the patient recovered in response to the defibrillation and/or chest compression procedures. Epinephrine was the most commonly used medication during treatment and was administered at doses ranging from 1 to 32mg (mean of 6.2mg). Atropine was used at a dose ranging from 0.5 to 4mg (mean of 1.5mg), and amiodarone was administered at a dose ranging from 150 to 1,200mg (mean of 369mg). Table 2 outlines the main characteristics of the 96 CPR procedures performed.

**Table 2:** Characteristics of the 96 episodes of cardiac arrest and cardiopulmonary resuscitation

Characteristics related to CPR	N (%)
CPR period (hours)	
Daytime: between 6 and 19 hours	62 (64.6)
Nighttime: > 19 hours and < 6 hours	34 (35.4)
Initial rhythm detected	
Asthya/bradyarrhythmia	28/13 (29.1/13.5)
Pulseless electrical activity	36 (37.5)
VT/VF	4/10 (14.6)
Unidentified	5 (5.2)
Medications administered	
Epinephrine	87 (90.0)
Dose of epinephrine > 3mg	43 (44.7)
Atropine	38 (38.5)
Sodium bicarbonate	28 (29.1)
Dobutamine	28 (29.1)
Amiodarone	
Defibrillation	31 (32.2)
Chest compression	94 (97.9)
Orotracheal intubation	22 (22.9)
Artificial pacemaker	5 (5.2)

CPR - cardiopulmonary resuscitation; CA - cardiac arrest; VT - ventricular tachycardia; VF - ventricular fibrillation.

The mean time at which CPR occurred was 1:07 PM (± 6:32 hours). The mean duration of patient hospitalization until the occurrence of CA was 10.3 days, with a median of 5 days. The mean duration of CPR procedures was 16.3 minutes, with a median of 11 minutes and range of 2 to 107 minutes. The other data on time periods between CPR procedures are outlined in table 3.

**Table 3:** Time periods related to the 96 episodes of cardiac arrest and cardiopulmonary resuscitation

CPR variables	Mean ± SD (median) value	Variation
Δt between CA and resuscitation (minutes; N = 66)	0.68 ± 1.3	0 - 9
Δt between CA and defibrillation (minutes; N = 6)	7.1 ± 5.1 (7.0)	1 - 15
Δt between CA and medication (minutes; N = 45)	2.5 ± 2.4 (2.0)	0 - 10
Δt between CA and OTI (minutes; N = 8)	4.8 ± 2.5 (5.0)	2 - 10
Duration of CPR (minutes; N = 96)	16.3 ± 16.7 (11.0)	2 - 107

CPR - cardiopulmonary resuscitation; SD - standard deviation; Δt - time period; CA - cardiac arrest; OTI - orotracheal intubation.

**Figure 2:**
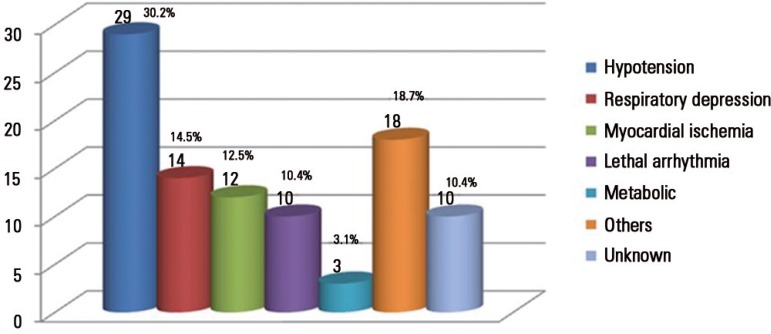
Causes of the 96 episodes of cardiac arrest, with the number of patients and their percentage. Others - hypoxia, orotracheal tube obstruction, pneumothorax and cardiogenic shock.

The most common cause of CA was arterial hypotension, followed by respiratory depression (Figure 2).

Gender did not correlate with age (age group ≥ 60 years); a diagnosis of myocardial infarction on admission; time or period of the day during which CA occurred; GCS score; time from admission to CA, resuscitation, defibrillation, the administration of medication, orotracheal intubation or the recovery of consciousness; the percentage of patients who underwent defibrillation; the percentage of patients who used epinephrine, dobutamine or intubation; or the percentage of patients who exhibited a return of spontaneous circulation. The mean duration of CA was 19.2 minutes in women and 13.5 minutes in men (p = 0.02).

Among the 89 patients, 45 (50.5%) exhibited some form of heart disease, and heart failure was the most common of these conditions. Heart disease correlated with age, age group ≥ 60 years, a diagnosis of acute myocardial infarction on admission and the use of dobutamine during CPR. These data are outlined in table 4.

**Table 4:** Association between heart disease and patient- and cardiopulmonary resuscitation-related variables

Variables	Heart disease	Non-heart disease	p value
Mean age (years)	60.6	53.6	< 0.001
≥ 60 years (Number and percentage of patients)	30 (66.6)	16 (36.3)	< 0.001
Acute myocardial infarction on admission	20 (44.4)	02 (4.5)	< 0.001
Glasgow Coma Scale	11.2	09.1	0.07
Daytime CA period	36 (75.0)	26 (54.1)	0.33
CA time (hours)	12.5	12.7	0.87
Δt between admission and CA (days)	8.3	12.2	0.42
Δt between CA and resuscitation (minutes)	0.8	0.5	0.72
Δt between CA and defibrillation (minutes)	3.2	5.0	0.38
Δt between CA and medication (minutes)	3.1	2.1	0.23
Δt between CA and OTI (minutes)	5.6	4.4	0.75
Defibrillation (Number and percentage of patients)	18 (37.5)	13 (27.0)	0.27
Intubation	13 (27.0)	09 (18.7)	0.33
Return of spontaneous circulation	35 (72.9)	35 (72.9)	1.00
Epinephrine use	43 (89.5)	44 (91.6)	0.73
Dobutamine use	14 (29.1)	06 (12.5)	0.03
Δt between CA and recovery of consciousness (hours)	13.5	21.4	0.28

CA - Cardiac arrest; OTI - Orotracheal intubation; Δt - Time period.

The comparison between patients diagnosed with acute myocardial infarction on admission and the remaining patients showed a significant association with age (66.5 versus 56.7 years, p = 0.01), the need for orotracheal intubation (45.8% versus 15.2%, p < 0.001) and GCS score (13.1 versus 9.0, p < 0.001). Acute myocardial infarction did not significantly correlate with any of the other variables.

Immediately after CPR, 26 (29.2%) patients died. The remaining 70.7% showed a return of spontaneous circulation, but 64.0% progressed with recurrent CA; specifically, 14.6% patients progressed within 20 minutes of the first CA, 24.7% progressed between 20 minutes and 24 hours after the first CA, and the remaining 24.7% patients progressed 24 hours after the first CA. The survival rate until discharge from the ICU or the coronary unit was 14.6%.

Eight patients were discharged from the hospital. However, 7 patients were followed up for 6 months after discharge (ages ranging from 57 to 66 years, 4 men). One patient was transferred to another hospital and thereby lost to follow-up. CA lasted for up to 6 minutes in these patients. Two of these patients had a CPC score of 2, and the remaining 5 patients had a CPC score of 1. One patient had a GCS score of 14, whereas the remaining patients had a GCS score of 15. Three patients continued with a CPC score of 1, 1 patient progressed from a CPC score of 2 to a CPC score of 1, and another patient worsened from a CPC score of 2 to a CPC score of 3 at the 6-month follow-up examination after discharge.

Two male patients died, one on the 24th day (while awaiting cardioverter defibrillator implantation) and another during the fourth month after hospital discharge due to a new CA. Thus, the 6-month survival rate was 5.6%.

The period of the day during which CA occurred was not associated with any of the following variables: age, gender, sedation condition, GCS score, defibrillation, intubation, CA time, time between procedures, the duration of CA, the return of spontaneous circulation, the use of medications and progression to death at the ward. Moreover the cause of CA, the initial rhythm of CA, the return of spontaneous circulation, CA recurrence and progression to death at the ward also did not correlate with any of the aforementioned variables, except for the return of spontaneous circulation and duration of CPR. The duration of CPR was 30.7 minutes for patients without a return of spontaneous circulation and 10.8 minutes for patients with a return of spontaneous circulation (p < 0.001).

## DISCUSSION

The setting of CA may affect patient survival because CPR should start earlier and patients are expected to show a return of spontaneous circulation if CA occurs in a hospital setting. Studies have reported better results for in-hospital CA in ICUs than for ward outcomes because the patient is being monitored, the events are promptly witnessed, and advanced life support is immediately available.^(12,13)^

The Brazilian Resuscitation Registry^(7)^ did not identify a higher survival rate among patients with CA in intensive care settings, in agreement with a study of 111 ICU patients that reported 100% immediate survival, although no patient survived to discharge.^(14)^ Other Brazilian studies of 150 and 452 patients showed 28%^(15)^ and 5%^(16)^ hospital discharge rates, respectively, although only 28% and 30.5% patients experienced CA in ICUs, respectively. Although immediate survival was 78.6% in the present study, the short-term and 6-month survival rates were low. This finding may be due to the severity of disease among these patients and the initial asystole rhythm and pulseless electrical activity, a predictor of lower survival.^(14,17,18)^ Furthermore, the in-hospital CA survival rates reportedly exhibit inter-hospital variability when adjusting for variables related to patient and hospital characteristics.^(5,19)^

Some characteristics of the patients in this study were similar to those reported in the literature. The ratio of men was also higher in cohorts from national and international studies, with rates ranging from 54% to 64%. Nevertheless, the mean age of the cohort in this study was similar to that of previous Brazilian studies and lower than that of the remaining studies, which included patients from different hospital settings.^(5,7,11,12,15,16,18-21)^ Patients with heart disease and acute myocardial infarction were older at the time of admission, which is consistent with the increase in the prevalence of these diseases with age.^(4)^ The prevalence rates of main comorbidities, including those of hypertension and diabetes, were similar to those reported in other studies.^(7,21)^ The most common admission diagnosis (24.7%) in the present study was acute myocardial infarction, whereas other studies reported rates of 17.7%^(7)^ and 19.5%.^(21)^ The percentages of patients using devices, including intra-arterial access and Swan-Ganz catheters, were similar to those reported in a previous study.^(21)^ However, the ratio of mechanically ventilated patients was higher in the present study (75.2%) than in the aforementioned cohort (27%) and a Brazilian study (20.9%).^(16)^ This difference may be attributed to the fact that only 55.8% of CAs occurred in the ICU or the emergency room in these studies, unlike the present study, which only examined CAs in this setting.

The time of day when CA occurs is a key factor. Most episodes of CA occurred during the daytime in the present study, which agreed with a study of a cohort of 86,748 adults, wherein only 32% episodes occurred at nighttime, resulting in lower survival.^(22)^

Arterial hypotension, the immediate cause of CA, was present in approximately 1/3 of patients in our cohort, and this incidence was similar to that published in previous reports.^(7,20,22)^ However, respiratory failure was less common (14.5%) in the present study than in previous studies (50.6%^(7)^ and 42%^(21)^), which may be due to differences in the setting of CA among these studies.

The initial rhythm at CA guides its management and affects patient survival.^(2-4)^ In hospital settings, the most commonly found rhythm is asystole, with rates ranging from 36% to 57%, followed by pulseless electrical activity, whose rates range from 16.5% to 39.3%.^(7,11,15-17,20,21)^ Although the incidences of these rhythms in this study are within these ranges, their limits widely vary. The populations in other studies derived from various in-hospital settings, including emergency rooms and wards in addition to coronary units and ICUs, and the monitoring of patients with CA may be delayed outside intensive care settings. In a population that exclusively experienced CA in the ICU, the main rhythm was ventricular tachycardia/ventricular fibrillation (38.5%), followed by asystole and pulseless electrical activity.^(23)^ Another contributing factor to CPR quality is team training; a survey conducted in a Brazilian tertiary hospital observed error rates of 66% and 79.5% in the identification of shockable rhythms pulseless electrical activity, respectively.^(24)^

In addition to high-quality chest compressions, some pharmaceutical drugs, including epinephrine and amiodarone, are indicated for cases of non-shockable rhythm and refractory ventricular arrhythmia, respectively.^(2,3)^ Nevertheless, atropine was used in more than 1/3 of CA episodes in this study, indicating poor adherence to guidelines.

Early defibrillation is a key factor for the survival of patients with CA due to ventricular fibrillation or pulseless ventricular tachycardia.^(25)^ In this study, defibrillation was performed in 32.2% episodes of CA for a mean duration of 7.1 minutes. The defibrillation rates were similar to those reported in previous Brazilian studies,^(7,16)^ albeit well below the rate recorded in the United States,^(20)^ which was 93% for a mean duration of 1.5 minutes (ranging from 0 to 30 minutes). A study of an in-hospital population with 910 episodes of CPR reported a mean duration of CPR of 4.2 minutes with a median of 2 minutes,^(26)^ which was reflected in the 37% in-hospital survival rate. Despite the intensive care setting of the present study, defibrillation was delayed.

The remaining Utstein-style time periods were not assessed for all patients, and this lack of data may also be observed in other published studies.^(7,15,16,18,20)^ Furthermore, a meta-analysis of CA in the ICU showed that most studies were retrospective,^(27)^ which also compromised the analysis of these data. In turn, the duration of CPR, a datum assessed in the entire population, was similar to the duration reported in other studies.^(7,15,16)^

A significant inverse association was detected between the duration of CPR and the return of spontaneous circulation. In a multicenter study of 64,339 patients, the duration of CPR also showed this association as well as an association with increased survival when compared with patients who underwent shorter (median of 16 minutes) and longer (25 minutes) CPR.^(28)^ Similarly, other studies have shown that shorter CPR durations were associated with higher immediate survival rates.^(7,13,16,18,23)^

Gender reportedly affects CA, and survival rates are higher in men than in women according to a multivariate analysis.^(29)^ However, neurological progression was better in surviving women than in men.^(30)^ In our study, survival did not differ by gender, although the duration of CPR was longer in women than in men, which favored neurological progression in women. Moreover, a recent study of 14,690 patients (36.4% women) with a mean age of 68.3 years showed no differences in survival between genders after adjusting for the Utstein-style variables. However, this study included patients with out-of-hospital CA.^(31)^ Conversely, another study showed an increased propensity for CPR among men with out-of-hospital CA.^(29)^ These contradictory records in the literature demonstrate the lack of epidemiological data on the subject.

Furthermore, recording all data recommended by the Utstein style is difficult because CPR requires focus and agility from the healthcare professionals involved in the maneuvers and that they are trained in recording such data. Accurately and completely filling out the record also implies that 1 fewer professional is available for CPR maneuvers. However, this standardized and comprehensive method contributes to the implementation of guidelines that improve the quality of care^(10)^ and consequently impact patient survival. To correctly fill out reports and subsequently analyze adherence to CPR guidelines, real-time documentation by an attending professional using a tablet may improve data quality and accuracy without compromising team performance.^(32)^

This method was not applied in the settings in which the present study was conducted. Therefore, only some Utstein-style variables were recorded in the medical charts, resulting in a non-standard report. The adoption of this method, combined with training the entire team, would enable implementing a high-quality care approach for CA and reach the time goals. The comparison between this group of study patients and a similar group after adopting the Utstein style will enable the assessment of care and ensure quality of care.

The present study has limitations, including the failure to complete the resuscitation report and the population size. Moreover, the study was conducted at a single center and in a public tertiary hospital and consequently fails to express differences that may exist between hospitals and regions.

## CONCLUSION

Asystole/bradyarrhythmia was the main initial rhythm detected in this population of patients with cardiac arrest in intensive care settings. The time from cardiac arrest to resuscitation was short, but defibrillation was delayed. Women had a longer resuscitation time than men. The prognosis was unfavorable, as evidenced by a low hospital survival rate, but neurological progression was good.
